# The commitment to a midwifery centre care model in Bangladesh: An interview study with midwives, educators and students

**DOI:** 10.1371/journal.pone.0271867

**Published:** 2023-04-10

**Authors:** Noor Islam Pappu, Ida Öberg, Ulrika Byrskog, Pronita Raha, Ratna Moni, Shaheen Akhtar, Priti Barua, Sujata Rani Das, Shipra De, Hosne Jannat Jyoti, Rezaur Rahman, Gita Rani Sinha, Kerstin Erlandsson

**Affiliations:** School of Health and Welfare, Dalarna University, Falun, Sweden; Hawassa University College of Medicine and Health Sciences, ETHIOPIA

## Abstract

**Background:**

Midwifery-led care is a key factor in reducing maternal and new-born mortality globally. In Bangladesh, only a third of births are attended by professionals and almost 70% of births occur outside healthcare facilities. Midwifery is a relatively new profession in Bangladesh and a midwifery centre care model has only recently been introduced. This study aims to explore the willingness within the healthcare system to support a greater role for midwifery centres in maternity services.

**Methods:**

Data were collected through individual semi-structured interviews with 55 midwives, midwifery educators and final year midwifery students. Two of the midwifery educators were principals of nursing institutes involved in the government’s midwifery leadership and considered as experts in the midwifery care system. The data was analysed using qualitative content analysis. The transcribed interviews comprised 150 pages. The study received ethical approval from the Directorate General of Nursing and Midwifery in Bangladesh.

**Results:**

One main category emerged from the study: “The foundations of a midwifery centre care model need to be strengthened for the sustainable implementation of midwifery centres in Bangladesh to continue”. Five additional categories were identified: 1) The midwifery centre care model is inaccessible for communities, 2) Striving for acceptable standards of care within a midwifery centre care model is not a priority 3) Respectful, woman-centred care is weak, 4) Community engagement with the midwifery centre care model is insufficient, and 5) The midwifery centre care model is not integrated into the healthcare system. These categories were supported by the identification of 11 sub-categories.

**Conclusion:**

The willingness to commit to a midwifery centre care model is not yet in place in Bangladesh. Advocacy, information, and education about the benefits of normal birth assisted by professional midwives is needed at all levels of Bangladeshi society.

## Background

Part of the United Nations’ third sustainable development goal (SGD) is reducing maternal mortality to less than 70 per 100,000 live births by 2030 [1]. Most maternal deaths occur in low-income countries and can be prevented when midwives attend births in an enabling environment [2, 3]. By scaling up midwifery interventions, maternal deaths can be averted [3]. Quality midwifery care has been shown to improve sexual and reproductive health, including increasing breastfeeding rates and improving early childhood development [4, 5]. The midwifery model of care differs from the medical model in the sense that it views pregnancy and birth as natural events and aims to minimize medical interventions around them by considering their social, cultural, biological, and psychological aspects [6]. A midwifery centre is a healthcare facility that provides care for women and new-borns, is rooted in midwifery philosophy and practices a midwifery-centred model of care. It is a home-like, shared space ensuring basic emergency maternal and neonatal care for all births. It is integrated within the healthcare system, aligning the level of care to optimal outcomes and is responsive to the needs of its community, with women’s experiences at its centre. The scope of care offered in midwifery centres varies greatly. In many low- and middle-income settings, midwifery centres often act outside government regulation. Suffer marginalization as a result, some are even forced to shut down [7]. This is despite the fact that midwifery centres, compared to hospital settings, are cost effective and do not pose a higher risk of maternal and new-born complications. Instead, research has shown that centres increase the number of spontaneous vaginal births and reduce medical interventions [8, 9]. Barriers to midwifery care in middle- and lower-income settings have been identified at the community level [10, 11]. Community engagement can facilitate more sustainable change by enabling interventions for maternal and new-born health that are grounded in socially and culturally accepted norms [12].

In Bangladesh, midwifery services have historically formed part of the nursing profession. However, in 2013 the government set up midwifery as a separate profession by introducing a midwifery training programme at nursing colleges [13]. By 2020 the country had approximately 1,100 trained professional midwives working in more than 300 health facilities. Nevertheless, only a third of births take place in a health facility or are attended by a healthcare provider [14, 15]. Giving birth in a health facility is getting more common and maternal mortality has started to decrease in Bangladesh [16]. The Bangladeshi government and other organizations are working together to educate midwives and improve midwifery-led care [15, 17]. However, the willingness of different parts of the healthcare system to accept midwifery was not, to our knowledge, investigated prior to the implementation of a model based on midwifery centres practicing midwifery-led care. Given that this model of care has only recently been introduced in Bangladesh this study aims to explore the willingness within the healthcare system to support a greater role for midwifery centres in maternity services.

## Methods

This study is an exploratory qualitative study investigating a topic that has not previously been studied in depth. Individual semi-structured interviews were carried out with Bangladeshi midwives, educators, and students. The data was analysed using qualitative content analysis according to Elo and Kyngäs [18]. Content analysis was deemed appropriate since it enables a qualitative approach to a large textual dataset. Further, content analysis is well suited for generating analysis-rich results within the often complex topics of nursing and midwifery [18].

### Participants and setting

This study interviewed 55 midwives, midwifery educators and third-year midwifery students. The participants were selected deliberately so that a variety of perspectives and experiences relating to the willingness to adopt a midwifery centre care model could be analysed. The participants varied in age, experience, education, and area of clinical expertise. Two of the midwifery educators were principals of nursing institutes involved in the government’s midwifery leadership and considered as experts in the midwifery care system. The participants were broken down as follows: *Midwives* (n = 27), mean age 34, mean 3 years of employment; *Midwifery educators* (n = 19), mean age 49, mean 10 years of employment; *Midwifery students* (n = 9), mean age 21, 0 years of employment. All participants had both theoretical knowledge and clinical experience of evidence-based midwifery care. The midwives had as a minimum qualification a three-year Diploma in Nursing and a one-year Diploma in Midwifery. Most of them had an additional six-month post-basic midwifery qualification which conformed to international midwifery training standards [3]. Those lacking this post-foundation training still had several years of experience providing evidence-based midwifery care. A small group of clinical midwives had completed a three-year direct entry Diploma in Midwifery programme, also conforming to international standards [3]. The midwifery students were all in the final year of this programme. Doctors and nurses without additional midwifery training and nursing educators were excluded from the study. Participants were recruited from across Bangladesh and were working or studying in 17 different healthcare facilities and 17 educational institutes. The healthcare facilities were located in both rural and urban areas, while the educational institutes were located in larger cities.

### Data collection

Dalarna University is a Swedish university that has, in partnership with the Bangladeshi government, developed an online MSc programme in midwifery. Between February and July 2020, 17 of the Bangladeshi students on this programme conducted the interviews (n = 55) that form the basis for this study. They prepared for this work by taking an advanced research methods course that included training in how to recruit study participants and conduct ethical semi-structured interviews. As part of their course, they read the article by Stevens et al. [7] that describes 13 operational standards for midwifery centres. Students and teachers worked together using these areas as the basis for development of a semi-structured questionnaire. To illustrate the operational standards, the questionnaire focused on five different areas. These were antenatal care, skin-to-skin care immediately after birth, early initiation of breastfeeding, delayed cord clamping, and upright birth positions [6, 19]. To capture the level of commitment within the healthcare system to a midwifery centre care model, the questions asked to what extent a midwifery centre would support these practice areas at the level of the individual, the family and the community. The operational standards that focused on administrative functions were used to investigate the relationship between midwifery centres and regulations and laws at a policy level [7]. The semi-structured questionnaire was developed in English and then translated into Bangla by a national programme coordinator skilled both in English and Bangla. The semi-structured interview guide is provided in a separate file.

The interviews were conducted at the different nursing institutes (n = 17) and healthcare facilities (n = 17) across Bangladesh where the 17 MSc students were working clinically under government orders, due to the Covid-19 pandemic situation in the country. The choice of healthcare facilities was based on where the 17 MSc students were working clinically under government orders due to the Covid-19 pandemic situation in the country. Course instructors asked the students to recruit two participants each to the study, one from a nursing institute and one from a healthcare facility. These 34 participants were the expected number in order to reach saturation. To assure full saturation of the five areas and the commitment of the individual, the family and the community, the students recruited an additional 17 more participants equally distributed from healthcare facilities and nursing institutes. An additional two principals and two midwives (n = 4) considered as experts in the midwifery care system were added to reach saturation of the policy level.

Each participant was given an invitation letter and a verbal introduction on what to expect during the interviews regarding the content. After giving their informed consent, an appointment for an interview was agreed upon. Due to the Covid-19 pandemic, interviews were conducted in both face-to-face settings and over the telephone. In order to prevent the transmission of the Covid-19 virus, face-to-face interviews took place only when the interviewers and participants had been vaccinated according to official government guidelines. During the interviews the interviewers and participants wore face masks, a suitable distance was kept, and windows were left. For the interviews that took place over the telephone, participants provided oral informed consent. Both interview formats took about 45–60 minutes and were audio recorded and transcribed verbatim into Bangla. They were then translated into English by the Bangladeshi midwifery educators themselves and double-checked by the national programme coordinator. None of the participants who gave their consent dropped out of the study.

### Analysis

The analysis of the collected data began with the preparation phase. Here the first and last author began by reading and re-reading the interview transcripts. With a sense of the data, they then provided instructions to the Bangladeshi students on how they should carry out their analysis of the transcripts. Students then, in the organizing phase, grouped together text parts, sentences and small paragraphs according to their similarities and differences. In the *organizing phase*, text parts, sentences, or small paragraphs, with the same meaning were grouped together based on similarities and differences in the text. These meaning units, as described by Elo and Kyngäs [18], formed the basis for the different sub-categories, which were in turn grouped together into categories at a higher hierarchical level. The content of the sub-categories and categories was agreed upon by the group. These showed that there were both barriers and facilitators to a midwifery centre care model. Using the 13operational standards for a midwifery centre model [7] as inspiration, five categories and 11 sub-categories have been operationalized by the coded material. These have been re-named to reflect their content. A main category was then created to summarize the commitment to a midwifery centre care model in Bangladesh. The analytical process is exemplified in Table 1.

**Table 1:** Example of the analytical process.

Main category	Category	Sub-category	Code	Meaning unit
The foundation for a midwifery centre care model needs to be strengthened if sustainable implementation of midwifery centres in Bangladesh is to continue	The midwifery centre care model is inaccessible for communities	Women must travel long distances to gain access to antenatal care or a facility birth provided by a midwife	Transportation to facilities with a midwife is lacking, unavailable or unsafe	To reach a midwife women have to confront internal transportation infrastructure problems. Some locations are remote and crossing a river, for example, can depend on the tidal volume, with some women having to wait a whole night before conditions suitable for motorised boat crossings are possible

### Ethical considerations

All of the participants received information about the content of the study and where to address any queries. They were informed that their participation was voluntary, that they could withdraw their participation at any time and that their identities would be anonymised at the group-level analysis. The study received ethical approval from the Directorate General of Nursing and Midwifery in Bangladesh (ethical clearance number 000170228).

## Results

This study revealed that the foundations of a midwifery centre care model need to be strengthened if the sustainable implementation of midwifery centres in Bangladesh is to continue. This main category was formed by the content of the five categories described below.

### The midwifery centre care model is inaccessible for communities

The statements from the participants in this study are related to structural factors such as suitable equipment for midwives, appropriate medicines, and accessible facilities. Responses indicate that these are often lacking thus making the midwifery centre care model non-accessible for many Bangladeshi communities. The participants indicate that this is particularly the case for poor communities in rural areas.

#### Women must travel long distances to gain access to antenatal care or have a facility birth provided by a midwife

Many women live far away from their nearest healthcare facility. Travelling to a facility is often impossible or unsafe because of poor transport infrastructure. Environmental obstacles, such as tidal rivers, can complicate access to midwifery care. One midwife describes this:

“Women have to face individual communication problem also, some locations are remote and depend on the tidal volume of the river, even some have to wait a whole night beside the river, after favourable situation the women came with engine boat” (Midwife 17).

Overall, the participants described transportation as a considerable challenge for accessing antenatal and other sexual and reproductive health services provided by midwives. The participants agree that home-visits by midwives needs to increase in rural areas.

The lack of midwives in Bangladesh contributes to this problem of accessing the midwifery centre care model. Although there are now a number of educated midwives working across the country, some participants explained that deployment has been slow, with posts not created in sufficient numbers, especially in remote areas. Despite this, participants note that midwifery education and the deployment of trained midwives is starting to make a difference and that without this new profession, accessibility to a midwifery centre care model would have been even lower.

#### Receiving equal care when reaching a midwifery facility is challenging

The insufficient number of midwives who work at the midwifery centres and the heavy workload of those that do make continuous support during labour and antenatal support for breastfeeding a significant challenge. Even if women are able to access a midwifery centre, participants describe long waiting lines for antenatal care, short opening hours in antenatal care, and a lack of seating and toilets in outpatient settings. “A midwife can’t stay beside only one mother as they have to do other works too, such as bed making, medicine and articles inventory, patient monitoring, carry out doctor’s orders” (Educator 4).

Poverty is also a barrier to proper treatment from midwives at a health facility. In Bangladesh, medical treatment often costs money. While some participants describe examples of subsidised midwifery care or subventions for medications, facilities often charged a fee for visits and check-ups. In addition, the antenatal care check-ups were in general not provided by midwives, but more often by doctors. One midwife says: “For complicated referral cases, money is needed. The poor patients cannot afford it” (Midwife 18). Instead, participants say that assistance for a home delivery from uneducated, traditional birth attendants, called Dais, is much cheaper than facility care or free of charge.

### Striving for acceptable standards of care within a midwifery centre care model is not a priority

The participants in this study say that the quality standards of a midwifery centre care model is not a priority among decision makers. They point out that the medical model and traditional hierarchal values are the standards of acceptability influencing healthcare practices. These give the midwifery centre care model and midwives a low authority and status.

#### Currently in Bangladesh the dominant model for maternity and neonatal healthcare practices is defined by medical doctors and nurses

This ‘medical’ approach to midwifery practices means that women are obliged to give birth in a lithotomy position, cord cutting is not delayed nor is there any support for breastfeeding. This culture prevails in many health facilities and educated midwives often feel they must give in to this medical approach. One midwifery student gives the following example:

“They [the doctors] will tell the mum that this [the lithotomy position] is good for the labour pain, the cervix dilation or if a caesarean section will be the outcome of the labour” (Student 8). Other participants describe how midwifery-led care has been introduced successfully in some clinical settings. However, most of them say that, in general, there is little interest for the midwifery centre care model among doctors and nurses. The acceptance of midwifery-led care is described as higher among health care providers in urban than in rural areas. Acceptance can vary even between healthcare providers at the same facility. One midwifery educator explains:

“Some healthcare providers are practicing the upright position. Some do not practice this. They practice the position of lithotomy. They lack evidence-based knowledge. Again, some healthcare providers do not practice knowingly. Because they cannot move away from their previous medical model habits” (Educator 3).

#### Decision makers are influenced by traditional hierarchical values in the development of quality standards for a midwifery centre care model

Decision makers create regulations, policy directions and determine the roles of healthcare providers that may create a conflict for midwives when they advocate a midwifery-led approach. The participants state that in Bangladesh, midwives are not able to work independently but must rely on ‘the orders and decisions taken by doctors. They describe incidents where midwives and midwifery students were actively held back from providing evidence-based midwifery practice in the labour ward. They describe being held back from counselling pregnant women in antenatal care. Several explanations are given for the reluctance of some healthcare workers to implement the midwifery model of care. The low status of midwives is one factor. Participants describe how the low status of women in Bangladesh only adds to the problems of acceptance facing a new profession. The participants also suggest that the high workload for doctors and nurses in antenatal departments and in labour wards makes them uninterested in new practices and more likely to fall back into old habits and routines. Participants also understand the low acceptance for the midwifery centre care model as the result of a lack of skills and knowledge among nurses and doctors that is then justified after hand by traditional hierarchal values. They state that many nurses and doctors are probably not aware of the benefits of certain evidence-based midwifery-led care practices, such as delayed cord clamping, or lack knowledge of how to provide respectful woman-centred care. One of the midwives summarizes the situation as follows:

“Patient overload might lead to tiredness and lack of interest to implement new procedures. So, perfect quality midwifery care is not provided. The women will meet with health care providers that are unwilling to learn new things and do new things in their clinical practice and this malpractice will then be taught to the midwifery students” (Educator 7).

### Respectful, woman-centred care is weak

Many examples of how midwives and midwifery students provide respectful and women-centred care are described by the participants in this study. They share how midwives work with individual birth-plans, providing information and choice, understanding and empathy, as well as giving support and promoting normal processes. However, this provision is still weak in many places.

#### Midwives are committed to respectful and woman-centred care that is in line with a midwifery model of care

Trained midwives in Bangladesh represent a new cadre of healthcare providers being deployed into the healthcare system. As experts in the provision of midwifery-led care, they can be seen as facilitators of a respectful approach to maternal care in Bangladesh. One midwife says: “The patients responded that before they did not receive good and respectful care in the hospital but now they did” (Midwife 17). Other midwives highlight the response that this respectful approach can cause within the healthcare system:

“The midwife must be prepared for reactions [from other health care providers] and still write up the birth plan and provide quality midwifery care. The midwife must explain respectful care and the woman’s fundamental human right of having a choice during pregnancy and childbirth” (Midwife 21).

The participants explained that educated women were gaining access to information online, making them more than ever aware of their human rights, as well as their rights during pregnancy and childbirth. They predict that for this group of women, practice in the labour room might change. They give examples of women who stood up for their rights while facing disrespect. Educator 3 says that “When a woman wants to stand up or labour in the upright position, they do it that way” (Educator 3).

The participants suggest that women’s own demands for midwifery-led care might have a role to play in shifting practice towards a midwifery centre care model supported by midwives. When respectful woman-centred care is practiced within a midwifery centre care model women and their families might start to prefer a hospital birth assisted by a midwife: “If we ensure privacy and trust in the facility, then hopefully community and family members will think that institutional birth is preferable” (Student 8).

#### Healthcare providers, sometimes including midwives, fail to provide respectful woman-centred care

The participants in this study acknowledge from experience that, in some maternal care contexts in Bangladesh, women’s rights are not always protected, and their choices are not always respected by healthcare providers. Woman-centred care according to the individual women’s unique needs and preferences are thus not always provided. Some participants have seen the harassment and mistreatment of women, their subjection at times to unnecessary interventions, such as caesarean sections, and a general lack of support for the natural processes of birth. Participants describe how disinformation, or simple lack of high-quality information from healthcare providers, can make women view, for example, bottle-feeding or caesarean sections as superior to natural processes. Thus, they do not get a chance to make an informed choice. The participants also give examples of how women can face disrespect when they request evidence-based care and wish to reject care according to facility routines. These findings suggest that health messages and services may not be adjusted to the women’s circumstances and needs. One of the midwives claims:

“The hospital does not provide friendly and quality health services. More than 60 to 70 of the patients stay in the labour ward where the allocated bed is 15. So, pregnant and new mothers do not receive good services. Not even their privacy can be maintained” (Midwife 17).

The participants go on to elaborate on the often insufficient environment of the healthcare facilities and how this can be a barrier for the provision of respectful care. They explain that it can often be hard to maintain privacy in the facilities. Labour wards are often crowded and noisy and do not provide space for women to move around during labour nor do they offer a calm environment for mother and new-born to lay the foundation for successful breastfeeding. Participants also point out that the facilities are often short basic equipment and lack basic infrastructural elements such as such as lighting, heating, water, and sanitation systems.

### Community engagement with the midwifery centre care model is insufficient

The participants state that family members and the wider community have an impact on the acceptance of a midwifery centre care model for the management of pregnancy, birth and antenatal care.

#### Women do not know about the benefits of respectful, woman-centred care

Participants understand the lack of respect and lack of women-centred care at the health facilities to be a reflection of Bangladeshi social values as a whole. They explain how gender inequality is widespread and that women face discrimination in every aspect of their lives. Further, they point out that many women, often uneducated and illiterate, are not aware of their right to consent or choose. They argue that the lack of respectful and woman-centred care that patients receive at health facilities makes community acceptance of their services more difficult. When women and their families experience disrespect, a lack of choice, or no consideration for their cultural expectations, it is hard to build trust for facility midwifery care. If a facility cannot show that it protects women’s human rights, that it encourages free choice or that it listens to women, community support for the model will not be forthcoming.

“The decision for seeking healthcare services in mainly made by male members of the household, husbands in particular. When husbands see that women are still treated badly at a health facility they do not want to pay for their wives to be treated there” (Midwife 17).

The participants’ state that gender inequality and strong traditions of collective decision making can be a real barrier for the development of a midwifery centre care model in Bangladesh. To them, traditional practices and community preferences stand in the way of evidence-based midwifery practices. The examples they provide include giving a new-born honey instead of colostrum, washing and wrapping the new-born instead of putting it skin-to-skin immediately after birth, and a reluctance to allow upright birthing positions or delayed cord clamping. Not taking part in antenatal visits or practicing homebirths without skilled professionals are also described as common practice in many communities. Other communities share the view that a caesarean section is superior to a vaginal birth. Participants view a lack of understanding within local communities as one reason for their reluctance to accept midwifery-led care and their continued support for traditional birth practices. They describe a lack of knowledge about the benefits of a midwifery model of care, as well as misconceptions about its practices. For example, they describe the belief that vitamins during pregnancy will result in a large baby and make a caesarean section necessary. They describe the idea that a new-born will get hurt if women adopt upright birthing positions. As one respondent explains:

“Relatives such as grandmother, grandfather hold the new-born baby after birth and keep it separate from the mother. They want to wash, dry, and keep the baby in their way. Sometimes, they give honey to the baby. Because of the belief that if the baby first eats honey, in the future the baby will talk sweetly and besides this, they do not know about skin-to-skin care and its importance and benefits. They have no interest in new things and follow traditional beliefs. They think of the cultural things they maintained, all of those for many years, which did not bring any harm” (Midwife 16).

Sometimes the participants offer a religious explanation for these community practices. For example, some describe how Muslim women in Bangladesh are not supposed to expose their breasts, thus limiting skin-to-skin contact and successful breastfeeding. Others state that for some communities, their faith means that they see no reason for taking part in antenatal or facility care, as they believe their fate is up to God. That said, other participants dismiss religion as an explanation: “There is nothing religiously said about this [skin-to-skin contact]. Lack of motivation in the community. The community people do not know or will not accept it” (Midwife 21).

The participants describe how women are not informed about their human rights and sometimes do not know that midwifery services exist. They report that female illiteracy and financial dependence makes women’s empowerment difficult. With this observation participants identify social problems as a structural obstacle preventing the wider acceptance of the midwifery model of care. While health education in local communities is one way forward, participants stress that midwives cannot overcome these social challenges alone. They emphasise that public awareness about women’s rights and their maternal health needs to increase. Educator 15 provides evidence of the holistic approach already being taken to engaging communities with the midwifery centre care model:

“To promote upright positions [during labour] in the family and at the community level, we emphasize people’s awareness, involve mass media, show films, newspapers, leaflets, posters, health education programmes, field worker training, healthcare provider training, clinical skilled birth attendant training, awareness programmes among community and village leaders” (Educator 15).

#### Women and families do not know about midwives’ commitment to respectful and woman-centred care

Many women continue to believe that midwives at a health facility cannot offer the same degree of maternal and new-born care as is provided by a “Dai”, the traditional birth attendant. Women and their families still believe that a health facility birth will lead to a costly and disrespectful treatment. In order to avoid delayed care in labour or an unnecessary caesarean section or episiotomy without consent and to give birth with a supportive and caring assistant, many women and families continue to choose a Dai. Participants point out that facility births do not accommodate cultural birthing preferences. Facilities only allow one birth companion, but most women wish to give birth surrounded by their entire family. Many women appreciate a supportive female birth attendance but facility births are often attended by male doctors. One respondent offers her suggestions for increasing acceptance of a midwifery centre care model:

“During admission in the hospital, no cost, no service charge, no waiting, all facilities under one umbrella, good staff behaviour, female health care personnel, food for the period of hospitalization, free cost of transportation. This nice treatment of women advocates for the services” (Midwife 27).

Men in the community or the family often decide whether the woman is allowed to take part in antenatal care or give birth at a facility. When the woman is under-age, this control is further reinforced by the fact that she has no legal right to decide. Thus, the high rates of child-marriage in Bangladesh are something the participants view as threatening access to a midwifery centre care model for women and girls.

Even with midwives’ theoretical education and practical simulations, the participants feel midwifery students and midwives often face challenges when they start working in real communities. New midwives sometimes lack good communication skills and find it difficult to adapt their knowledge to cultural contexts. Young students can have a hard time navigating the contradictions between the theory they learned in college and the practices they encounter in rural communities. They are often confused by the contradictions they encounter between what people say and what they can see. This makes it hard for them to know what to believe and, by extension, to remain consistent in their advice to women. Midwife 4 elaborates on how this ambivalence can weaken community acceptance:

“That’s why students cannot counsel properly, and community people do not get a clear message from the midwifery students. As a result, it might be reduced community acceptance of students, midwives and their practice” (Midwife 4).

The participants believe that midwives have an important job to play in Bangladesh. They feel midwives are there to “empower women”, as well as to influence and inform them and the entire community about maternity best practice. They are acutely aware that local practices and values need to change if there is to be a greater commitment to a midwifery centre care model. One participant feels: “We have failed to give the message at the grass-root level, because midwifery education is new in Bangladesh, and we have not enough midwives” (Student 6). Her comment points to an additional obstacle to midwifery’s acceptance: as a new and still marginal profession, midwives need help building trust in their communities. Some participants suggest that members of hospital management or other leaders could take part in health education sessions to enhance the message of the midwives.

#### Strong collective traditions could enable the change to a midwifery centre care model

Some participants state that some of the communities in which they work are more aware than ever about the advantages of midwifery care. They see this as a positive step and a possible mechanism for future change, even though, the participants in this study agree that implementing a midwifery centre care model comes with several challenges on a community level. Dais and older women as well as imams and village doctors are described as key stakeholders in the upholding of traditional practices in local communities. Women often share the values of their family and community and follow their suggestions. Changing the views of these local influencers is critical for the wider acceptance of a midwifery-led care model.

The participants describe initiatives that are currently taking place to increase community awareness and acceptance of a midwifery centre care model. They state that midwives or midwifery students arrange community health education sessions where they provide information on, for example, early initiation of breastfeeding, delayed cord clamping and the warning signs for preeclampsia. One midwife explains:

“After identifying the level of awareness and current practice, the midwifery students go door-to-door in the communities. They invite and arrange health education sessions, short role-plays, and posters. In this way, the community people will be able to learn about the importance [of breastfeeding]” (Midwife 16).

When talking about community engagement, the participants express attitudes and give suggestions that are in line with a midwifery centre care model. For example, they outline the importance of considering cultural beliefs in education sessions and give suggestions for working with stakeholders to improve women’s health. At the same time, they put the individual woman’s rights before family tradition. These perspectives should encourage greater community acceptance of midwifery-led care and contribute to women’s empowerment. In a small number of cases though, participants do talk about communities, families, and women in a negative way, as “refusing to learn” or, on one occasion, as “lazy” or “ignorant”. Such frustrations are understandable, given the resistance that midwives encounter in their everyday work. They indicate that midwifery education is still a work in progress and that the balance between cultural sensitivity and evidence-based practice must be delicately negotiated. Encouraging midwives to see collective traditions as a potential source of changed attitudes within local communities is a challenge for the midwifery leadership and others advocating for a midwifery centre care model.

### The midwifery centre care model is not integrated into the healthcare system

According to the participants in this study, dedication to the midwifery centre care model is low within the Bangladeshi healthcare system, at both institutional and governmental levels.

#### Guidelines for quality midwifery care do exist at a policy level, but these have not yet reached healthcare facilities

The Bangladeshi government has produced written policy documents committing the healthcare system to promoting midwifery practices and values that are in line with international midwifery standards. According to the participants in this study, this commitment is not evident in health facilities. There is, at times, little sign of evidence-based midwifery practices as well as respectful, women-centred care. One respondent gives an example:

“Every woman has the right to be free from harm and ill-treatment, every woman has the right to informed consent and to be respected for her choices and preferences, which includes a birth companion. But at the policy level, this has not been circulated to the institutional level. Proper circulation about respectful maternity care is needed” (Midwife 10).

Healthcare facilities may be aware at an institutional level about the government’s commitment to a midwifery centre care model, but participants describe everyday scenarios where early initiation of breastfeeding or delayed cord clamping are not being recommended. Health facility staff who lack training in midwifery-led values continue to drive routines which make midwifery-led care possible. One midwife says:

“If a woman wants support to initiate breastfeeding immediately after birth, she might not get it, because the routines at the institution will not allow [it]. She is not taking decisions about her own body and her child’s wellbeing when delivering in a health facility or hospital” (Educator 5).

#### Midwives are not sufficiently represented on government bodies responsible for midwifery which is preventing acceptance of the midwifery centre care model

When identifying ways to extend acceptance of the midwifery centre care model in Bangladesh, some of the participants take a top-down approach. They suggest that support for midwifery-led care is dependent on a culture of gender equality, something which must be sponsored at the highest levels. One midwife says:

“Stigma and the perception of both clients and providers that vaginal birth is of less value than caesarean section births must be dealt with from the top level. Gender inequality, (…) why can a man decide over women and girl’s bodies” (Midwife 2)?

That said, participants do note that the implementation of national guidelines is more successful in health facilities located in urban areas and that awareness and skills training is on the rise. Many credit the work of local NGOs who provide education sessions and support for midwifery-led values at health facilities. Many point to the importance of practical and”hands on” training as a way to increase acceptance of the midwifery centre model of care. “We know that midwives play a key role, but it is a lack of motivation to implement respectful maternity care at an overall and structural level” (Educator 17).

The participants describe how policymakers are often unaware of the importance of midwifery-led care. Midwifery educators, for example, often describe feeling a lack of support from the government and its agencies for sustaining the midwifery workforce. One midwifery educator shares her perspective on this: “The government doesn’t seem to support our work. They never give us enough money and are slow to implement our recommendations” (Educator 19).

In particular, participants point out that government monitoring and evaluation of midwifery care in health facilities is infrequent. They also note that the recommended tools for assessing and accrediting student midwives out on placement are not sufficiently used. The participants state the value of the practical help and expertise Bangladesh has received from a number of external NGOs but argue that if sustainable quality maternal health care is to thrive in Bangladesh, the government and its agencies must take responsibility for operating and monitoring the midwifery centre care model. Healthcare providers on all levels still lack knowledge of the practices and values of a midwifery centre care model. The systems for developing the competencies of nurses doctors and other healthcare personnel are still insufficient. Midwife 11 says: “There is still no systematic way that doctors and nurses can learn more about midwifery-led care. Spreading information about midwifery is still very ad hoc and random” (Midwife 11).

## Discussion

This study aims to explore the willingness within the healthcare system to support a greater role for midwifery centres in maternity services. This from how Bangladeshi midwives, midwifery educators and final year midwifery students perceive the commitment of the healthcare system to adopting a midwifery centre care model in Bangladesh. Their observations suggest that the foundations of the midwifery model of care need to be strengthened if the implementation of accessible, respectful, sustainable, woman-centred, and community-engaged midwifery centres are to be fully integrated and accepted in the Bangladeshi healthcare system.

A review of the existing literature on birth centres indicates that spontaneous vaginal birth rates and perineal integrity were higher for women beginning their care in birth centres utilizing a midwifery centre care model than those receiving traditional hospital care. Rates of caesarean births were lower, there were fewer severe maternal outcomes and no reported maternal deaths. Women were satisfied with the comprehensive and personalized care they received [20]. This review was, however, based on 32 studies from the United States, England, Sweden, Denmark, Australia, Canada, Scotland and Germany. These settings are quite different from the situation in Bangladesh. Whereas accessibility to a facility adopting a midwifery centre care model is high in these countries, it is low in Bangladesh. Low access to midwifery-led care [10] is common in low- and middle-income countries. In 2017, one third of the global population lacked access to even the most essential health services [2]. However, poor access to midwifery services is affected by several factors [10]. The main factor in Bangladesh affecting accessibility to a midwifery centre care model is the lack of trained midwives [21–23]. On the one hand, only a small number of midwives are being educated. On the other, the failure of many health facilities to employ midwives means there are few job opportunities for trained. The State of the World’s Midwifery report in 2021 [23] mentions the lack of midwives trained to international standards. It also pointed out that extensive time spent on non-midwifery tasks increased midwifery workloads and left limited time for essential midwifery tasks. Similar issues confront the projected upscaling of midwifery-led care in Bangladesh. With a full complement of internationally trained midwives who are fully integrated into the existing healthcare system and supported by a fully functioning network of referral and transfer mechanisms to specialist care, midwives could avert a total of 83% of all maternal deaths, stillbirths, and neonatal deaths [3, 24] also in Bangladesh. In Bangladesh, Byrskog et al. [22] has outlined how the lack of cultural understanding and the lack of a supportive environment for midwives can affect quality midwifery care and increase work-related stress [21, 22]. Zaman et al. [25], however, find that Bangladeshi midwives have reasonable workloads.

Advocating a midwifery centre care model can appear to support yet another institutional approach to the treatment of childbirth. In Bangladesh, health facilities and the midwives who are based at them are often far away. Insistence on facility appointments and births can require long journeys which participants in this study describe as unsafe or arduous [10]. This lack of transportation infrastructure can create a sense of anxiety about being able to get to a facility in time to give birth. Seeing birth as an unpredictable event, and not planning ahead, many women end up having a homebirth [10]. The participants in this study point out that an important cultural factor behind homebirth is that health facilities only allow one birth companion. This means that women cannot have their entire family around them when giving birth, as is customary. The importance of family being present at Bangladeshi births has previously been described [9]. Family interaction and involvement in birth should be a feature of respectful maternity care [26]. Another factor contributing to a reluctance to have health facility births is that women must pay for their care. Homebirths accompanied by a traditional birth attendant are much cheaper or free of charge. Women perceive going to a health facility as something that is done only in extreme health crises. For most of them giving birth is a natural part of life and something that can be easily managed at home [10].

The well-known expression “too little, too late and too much, too soon” describes the obstacles to the expansion of evidence-based, respectful maternity care worldwide [27]. The results of this study illustrate the point. The opportunities midwives have to work according to the midwifery centre care model are restricted by the conflicting opinions of other healthcare providers. One example is when doctors advise women to give birth in the lithotomy position. According to the participants in this study, the lack of knowledge amongst nurses and doctors about evidence-based midwifery care can impede the implementation of a midwifery centre care model. According to previous studies with midwifery students in Bangladesh, doctors think they know better than midwives and nurses [21, 22]. The participants in this study state that educated midwives are committed to respectful and women-centred care and know that there are international guidelines, issued by the ICM, which govern the way they work. [28]. However, educated midwives in Bangladesh are too few and the patient flow is too high. Even if they and other caregivers are committed to the highest quality care, they often fail to do so. Volume and the workplace routines which have been developed to manage these mean that midwives and other care providers are, to some extent, restricted in their actions. Some midwives have even reported cases of malpractice [21]. Moreover, many midwives do not feel safe at their workplace or when travelling. Being young women, these insecurities can have a negative impact on their competence performances [22]. The low status of midwives in Bangladesh is another factor accounting for the low implementation of a midwifery centre care model. One cause could be low status of women in general [21]. The findings suggest that not everyone in Bangladesh even knows that midwives exist as a profession. Confusion could be a result of the fact that in Bangladesh both traditional birth attendants and educated midwives are called “dai”. In India, however, educated midwives are now given the title of “Nurse Practitioner Midwife” [29]. Strong collective traditions can be both a barrier and a facilitator to the implementation of a midwifery centre care model. Bohren et al. [10] writes that many local people do not believe that midwives can provide better care than a dai. Because gender inequality is endemic throughout Bangladesh [21, 22], young female midwives can find autonomous decision making difficult. If they lack the skills to make decisions, how can they act authoritatively and with confidence? If people in general do not know what midwifery education involves in terms of knowledge and skills, and therefore do not have faith in the young female midwife, as one article [21] has depicted it, what then is needed to strengthen young midwives? Filby et al. [11] writes that lack of confidence can lead young midwives doubt their own abilities. If other healthcare providers at their workplace and people within the wider community do not trust them to act correctly [22], who can give them the support they need to become both competent and confident? Due to the low status of women, a woman’s family and community can have a great impact on her treatment during pregnancy, birth, and new-born related care. It is often male family members, mostly the husband, who decide whether the woman is allowed to take part in antenatal care or give birth at a facility. This means it is the men and the wider community who need to be reached with the message of a midwife’s scope of practice and her competence in facility-based births. Bohren et al. [10] writes that the husband plays an important role regarding the location of a birth. As long as they resist facility births, the midwifery centre care model in the Bangladesh healthcare system will be met with resistance. Bangladeshi maternity care still has improvements to make. Women’s rights are not always protected and healthcare provides do not always respect women’s choices. Sometimes, women do not want to receive care according to facility routines, for example due to cultural contexts, but then they faced disrespect from medical staff. Bohren et al. [30] describe cases where women had to endure both verbal and physical abuse at a health facility. Where women were afraid, they might be mistreated at a health facility [10]. In this study [10], poor quality information from caregivers often prevented women from making informed choices. This is an area that needs to change if women are to make midwifery care their desired choice. This argument becomes, in turn, the justification for working to extend a midwifery centre care model in Bangladesh. However, a lack of respectful and woman-centred care makes community acceptance difficult [30]. When women experience disrespect, lack of choice, or no consideration for cultural expectations it is harder to build trust in a midwifery-led model. Despite this, some women already consider a health facility as the safest place for them to give birth [3]. If the midwifery centre care model is to gain a critical foothold in Bangladesh, it needs to be promoted among not just women, but also among those other influential actors: the local community and healthcare providers.

### Strength and limitations

Using postgraduate students to carry out the interviews upon which this study is based can be seen as a credibility risk. This was addressed by providing the students with interview training via an advanced methodology course with a workload equivalent of 200 hours. This course included rigorous training by Dalarna University lecturers in interview techniques and ethics as well as a full explanation of the context for the study. Each of the 13 questions and follow up questions were explained and discussed. The fact that each student carried out their interviews on their own may have resulted in slightly different interpretations of the interview guide and thus potentially different applications of the questionnaire. This may have influenced both the dependability and the credibility of the study. Credibility and dependability are criterion for evaluating trustworthiness in qualitative studies. Credibility refers to confidence in the truth of the data and dependability refers to the stability of the data over time and in different conditions [31]. To reduce the risk of personal bias, this study has followed the Standards for Reporting Qualitative Research [32]. The authors have made an effort to provide readers with a complete description of the data gathering and analysis process so that accurate conclusions can be drawn. The authors have, to the best of their ability, used the methodology correctly and have not made any methodological mistakes. The interview recordings were transcribed verbatim into Bangla by the student interviewers themselves. They were then double-checked by the national coordinator of the MSc programme who had been briefed on the project so as to avoid misinterpretations. The analysis was carried out using a step wise process involving refraining from pre-judgements and using the pre-understandings of some of the team to inform discussions within the wider research team which could benefit the validity of the results [31]. The authors were able to reveal the views of the participants by following Elo and Kyngäs [18], where purposive sampling is used to get a variety of perspectives on a topic [31]. The qualitative description which this study provides is not sufficient to understand the midwifery centre care model in Bangladesh. However, despite its limitations the content of this qualitative article draws some important conclusions about the challenges facing the introduction of a midwifery centre care model in a country where the midwifery profession and its philosophical foundations are still only newly introduced.

## Conclusion and clinical implications

The commitment to a midwifery centre care model is not yet firmly in place in Bangladesh. This is due systemic problems, such as gender inequality, and the difficulties of convincing the hearts and minds of key influencers, such as medical personnel and family and community members. If Bangladesh wishes to see midwifery-led care gain a serious foothold in the country, medical and transport infrastructures must be in place and respectful professional care must be provided. Both women and midwives need a safe environment where adequate care can be given and received. Advocacy, information and education about the benefits of a normal birth assisted by a professional midwife are needed at all levels in Bangladeshi society before women and their male family members will begin to demand a midwifery centre care model. The need for advocacy, information and education about midwifery centre care model, as the clinical implications of this study point out, must go hand in hand with an overarching strategy to reduce gender inequality promote the wider acceptance of midwives in society.

### Suggestions for further research

As far as we are aware, there is very little research on the degree of commitment members of the healthcare profession believe exists within the Bangladeshi population for a midwifery centre care model. All of the participants in this study were women because midwives, midwifery educators and midwifery students are women in Bangladesh. An investigation of the attitudes of male medical professionals whose work brings them into contact with the care and management of reproductive and birth processes would be welcome complementary view. How doctors in Bangladesh view the new midwifery profession and its aspirations to promote a midwifery centre care model would do much to balance the to date highly gendered perspective on this vital and important question.

## Supporting information

S1 File Semi-structured questionnaire English version. (DOCX)
