# Supplementary material for: The commitment to a midwifery centre care model in Bangladesh: An interview study with midwives, educators and students
Source: PLoS One. 2023 Apr 10;18(4):e0271867. doi: 10.1371/journal.pone.0271867 (PMC10085017; doi:10.1371/journal.pone.0271867)
Supplement: S1 File — (DOCX) [file pone.0271867.s001.docx]

**Semi-structured questionnaire**

**WOMAN focused Standards**

1 There is an expressed institutional commitment that: every woman and newborn will be treated with respect and dignity, no woman or newborn is subjected to mistreatment; including physical abuse, sexual abuse, verbal abuse, discrimination, neglect, detainment, extortion, denial of services, and no care or procedures done without informed consent.

- Please tell me your experiences of this topic.
- Please give me examples for instance from antenatal care, on skin-to-skin care immediately after birth, early initiation of breastfeeding, delayed cord clamping, or dynamic birth positions

2 Every woman has access to her health information and receives information about her care, the reasons for interventions and outcomes are clearly explained, so the woman is able to make informed choices about the services she receives.

- Please tell me your experiences of this topic.
- Please give me examples for instance from antenatal care, on skin-to-skin care immediately after birth, early initiation of breastfeeding, delayed cord clamping, or dynamic birth positions

3 Communication with users is respectful. For example, mothers are addressed by name, unknown staff members identify themselves, the roles of various staff members are clearly communicated.

- Please tell me your experiences of this topic.
- Please give me examples for instance from antenatal care, on skin-to-skin care immediately after birth, early initiation of breastfeeding, delayed cord clamping, or dynamic birth positions

4 There is shared decision making for all services related to pregnancy, birth and newborn care

- Please tell me your experiences of this topic.
- Please give me examples for instance from antenatal care, on skin-to-skin care immediately after birth, early initiation of breastfeeding, delayed cord clamping, or dynamic birth positions

5 Every mother is offered an orientation to the birth center’s model of care and a tour of the facility

- Please tell me your experiences of this topic.
- Please give me examples for instance from antenatal care, on skin-to-skin care immediately after birth, early initiation of breastfeeding, delayed cord clamping, or dynamic birth positions

6 There is a mechanism for home visits, either routinely or as needed.

- Please tell me your experiences of this topic.
- Please give me examples for instance from antenatal care, on skin-to-skin care immediately after birth, early initiation of breastfeeding, delayed cord clamping, or dynamic birth positions

7 User confidentiality is respected

- Please tell me your experiences of this topic.
- Please give me examples for instance from antenatal care, on skin-to-skin care immediately after birth, early initiation of breastfeeding, delayed cord clamping, or dynamic birth positions

8 The environment offers adequate privacy based on user preferences

- Please tell me your experiences of this topic.
- Please give me examples for instance from antenatal care, on skin-to-skin care immediately after birth, early initiation of breastfeeding, delayed cord clamping, or dynamic birth positions

9 The midwifery center participates in interactive community activities

- Please tell me your experiences of this topic.
- Please give me examples for instance from antenatal care, on skin-to-skin care immediately after birth, early initiation of breastfeeding, delayed cord clamping, or dynamic birth positions

10 Every mother is informed about the benefits of supporting physiological processes such as drug-free comfort and pain relief methods to support normal labor

- Please tell me your experiences of this topic.
- Please give me examples for instance from antenatal care, on skin-to-skin care immediately after birth, early initiation of breastfeeding, delayed cord clamping, or dynamic birth positions

11 Every mother is informed of the benefits and encouraged to have continuous physical support, by someone of her choosing- including family and traditional healers- to accompany her during labor and delivery.

- Please tell me your experiences of this topic.
- Please give me examples for instance from antenatal care, on skin-to-skin care immediately after birth, early initiation of breastfeeding, delayed cord clamping, or dynamic birth positions

12 Each mother is asked about in her cultural traditions and spiritual expectations pertaining to her pregnancy, delivery, and postpartum care for herself and her newborn and if safe, supported.

- Please tell me your experiences of this topic.
- Please give me examples for instance from antenatal care, on skin-to-skin care immediately after birth, early initiation of breastfeeding, delayed cord clamping, or dynamic birth positions

13 Every woman receives support to strengthen her capability during childbirth including childbirth education, and comfort measures.

- Please tell me your experiences of this topic.
- Please give me examples for instance from antenatal care, on skin-to-skin care immediately after birth, early initiation of breastfeeding, delayed cord clamping, or dynamic birth positions

**PROVIDER focused Standards**

- Thinking of health care providers, the Bangladesh society, the individual, the family and community what do they think of midwifery led care at Midwifery Centers?

**Community-Administrative focus Standards**

- Thinking of administration, regulations, laws and policies at health care and education levels, what do the Bangladesh society, the individual, the family and the community think of midwifery led care at Midwifery Centers?
